# Monitoring a 28.5 m High Anchored Pile Wall in Gravel Using Various Methods

**DOI:** 10.3390/s20010080

**Published:** 2019-12-21

**Authors:** Ricardo Moffat, Pablo Parra, Miguel Carrasco

**Affiliations:** Faculty of Engineering and Sciences, Universidad Adolfo Ibáñez, Av. Diagonal Las Torres, 20700, Peñalolén, Santiago 7941169, Chile; Pablo.Parra@uai.cl (P.P.); Miguel.carrasco@uai.cl (M.C.)

**Keywords:** deep excavation, anchored wall, monitoring, pile displacement

## Abstract

Horizontal displacements of a multiple-anchor pile wall in a 28.5 m deep excavation using the top–down construction method have been monitored using optical fiber (Brillouin optical time-domain reflectometry (BOTDR)), strain gauges, inclinometers, and a topographic survey. This work presents a comparison between these different techniques to measure horizontal displacements in the pile at several stages of the soil excavation process. It was observed that displacements can be separated into two components: Rigid body motion and pile flexural deformation. Measurements using optical fiber and inclinometers are considered the most adequate and easy to install. A numerical model allows us to evaluate the influence of earth pressure on the estimated horizontal displacements. It is shown that using soil pressure on the wall given by p = 0.65Kaγh, on a simplified modeled wall, provides a close deduction of horizontal displacements compared to observed values on the field.

## 1. Introduction

The monitoring of soil slopes [1,2] and retaining walls [3] was shown to be useful for identifying the main factors that contribute to safe design. Urban building excavations require stable support systems that can provide lateral support, reducing lateral deformations of these walls or piles to avoid or decrease their impact on the surrounding structures. As the walls or piles get larger, they require additional support systems such as anchors. In fact, it is currently common to use pre-stressed ground anchors using either tie rods or cable strands. As these elements are pre-stressed at the same time the excavation advances, most of the displacement during construction could be prevented.

The geotechnical design frequently uses the limit state approach to check the adequacy of the structure against failure or a serviceability limit state. Then, the structure is designed to satisfy a required factor of safety and deformations that are below serviceability limits depending on the type of structure. The adequate assessment of the safety factor, and expected deformations, need to have an adequate characterization of the soil properties (strength parameters and deformation modulus) and reasonable theoretical or numerical methods to predict the loads over the retaining system.

The design of a multi-level anchored wall depends on factors such as [4,5] soil resistance and deformation modulus; flexibility of the wall; necessary displacements to develop earth pressure; and applied anchor loads. The stress distribution behind a wall depends on the construction method or stages, anchor pre-stressing, and relaxation. It is not always accurate to assume that a fully active condition can be reached in this type of wall, as pre-stressed anchors do not allow free deformation and displacement of the soil as required to reach the active conditions. Higher lateral stresses are expected in zones closer to the anchor location as the anchors produce a condition that is closer to the passive state of the soil.

Different studies on deep excavation have been performed using numerical modeling or through monitoring the displacements or strains of walls. A 14.7 m deep excavation was modeled by Hou et al. [6] using 3D finite-element analyses, finding that soil anisotropy has a significant effect on wall deformation. Schwamb et al. [7] successfully used fiber optic monitoring in a 73 m deep circular excavation with minimal disruption of the wall construction process. Different instruments were used on a monitoring program in a deep excavation sustained by an anchored diaphragm wall by Ene et al. [8]. Lam et al. [9] performed a series of centrifuge models of deep excavation in soft soils. They found that the trend lines given by Clough et al. [10] matched their results and that a small strain stiffness is very important in the deformation mechanism. Nikolinakou et al. [11] described a 20 m diaphragm wall in sand with pre-stress anchors and showed good agreement between the measured and modeled wall deflections and forces observed in the study.

This research is based on a deep excavation 28.5 m deep, extending 65 m in the West-East and 90 m in the North-South directions, that was monitored in Santiago, Chile (located between 33° and 34° S at approximately 100 km from the coastline). Santiago’s basin is mainly composed of alluvial and fluvial sediments originating in the Maipo and Mapocho river basins. In most places, the gravel deposit has a thickness over 100 m. Santiago gravel is composed of boulders usually less than 20 cm in diameter in a matrix of soil that includes silty gravel to silty sand and some clayey lenses. Undisturbed soil samples have been tested in this material with samples of 1 m in diameter, as shown in detail in [12]. Friction angles for these gravels have been found above 50°. The deformation modulus has been measured using a large triaxial test on undisturbed soil samples and also using loading plate tests. Results show an increase in deformation modulus of the soil, from 50 MPa at the surface to 300 MPa about 40 m deep [13].

The pile wall consisted of 1 m diameter pre-bored piles made of cast-in-place reinforced concrete with a 3 m center-to-center spacing. Each pile had three pre-stress anchors; each anchor consisted of six steel cables (ASTM A416 GR270). This noncontinuous pile wall was monitored using different measurement techniques to compare its performance on-site. Numerical modeling was performed in order to carry out a parametric study of the influence of the applied loads on the deduced wall displacements.

## 2. Materials and Methods

The economical and safe construction of excavation support systems considers different design methods, complex geotechnical conditions, and numerous design software. A combination of theoretical and numerical models, empirical methods, and engineering experience is very common in practice. Excavation load stability and control of deformation are two key aspects of the design. The design of multiple anchor retaining structures requires an apparent earth pressure to be considered, but this pressure depends on a complex interaction between loads in anchors and soil condition. Monitoring the displacement of actual projects is essential to improve our design methods and assumptions; furthermore, without a monitoring system, the contract team has no early warning to deal with unexpected occurrences.

Slope indicator systems (inclinometers), strain gauges, surveying techniques, and, most recently, different types of optical fibers are being used to monitor excavation displacement and forces in structural members. In this work, different sensors or methods were used on the field to measure the displacements and strains of a noncontinuous anchored wall (or piles). This type of discontinuous piling support is frequently used in Santiago for temporary deep excavation as the soil consists of stiff gravel with a deep water table. Piles were constructed previous to excavation using a 1 m diameter pile drilling machine. Piles were separated (center to center) by approximately 3 m. A thin layer of shotcrete was placed between the space left by the piles to avoid boulders falling over construction workers. Three anchor lines were installed sequentially during excavation. Each line of anchors was pre-stressed with a force between 1000 and 1300 kN. The following sections present the main characteristics of the instruments used to monitor these piles.

### 2.1. Inclinometer

**Figure 1:**
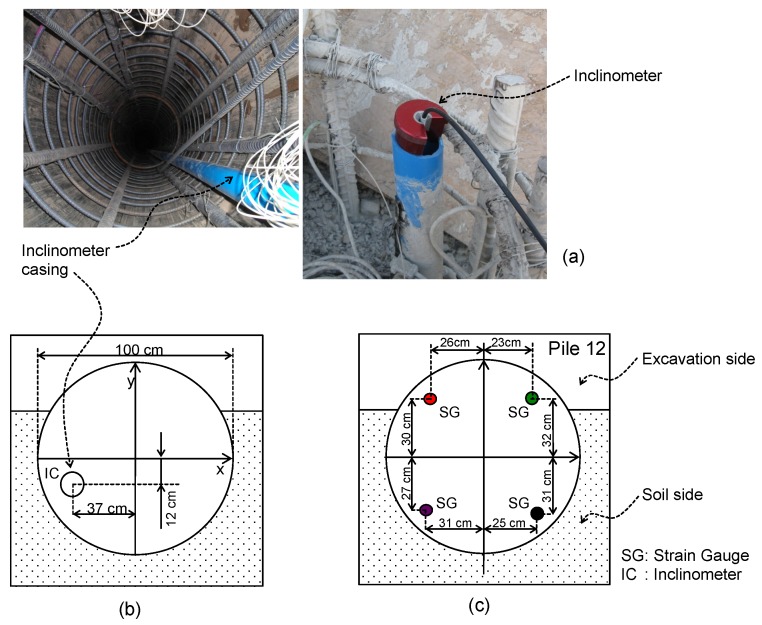
(**a**) Inclinometer inside casing installed in pile. (**b**) Installation and location of the inclinometer casing. (**c**) Installation and location of the strain gauges.

An RST retrievable inclinometer has two accelerometers in its interior that allow the inclination to be estimated in two perpendicular directions with respect to the vertical position. The necessary casing (external diameter 7 cm) was installed on the field during the pile construction, as shown in Figure 1. The inclinometer was lowered gradually inside the casing, and measurements were taken every 1 m with a probe 71 cm long and 2.54 cm in diameter. Data were transmitted to the surface by Bluetooth and saved by the data acquisition system (iPAQ hx2410) located on the surface. Table A1 shows the main characteristics of the casing, and Table A2 shows the main characteristics of the inclinometer. The displacement error was 2 mm per 25 m, the measurement range was ±38°, and the error was estimated to be lower than 0.01°. An initial or base measurement was carried out when the pile was constructed, but before the excavation started.

### 2.2. Strain Gauges

**Figure 2:**
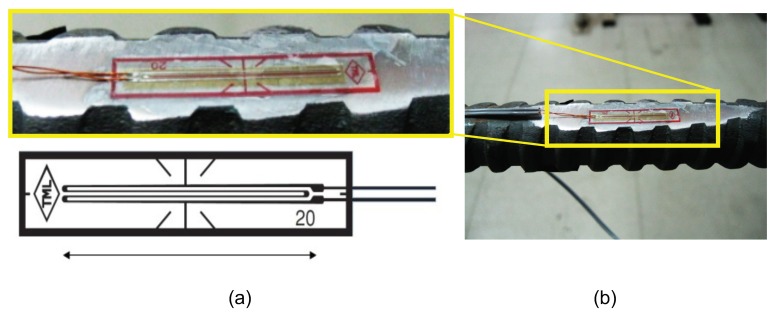
Photo of installed strain gauge ion reinforcement of piles (photo of strain gauge PFL-20-11).

TML strain gauge model PFL-20-15 (gauge factor of 2.12 + 1% and thermal expansion coefficient of 118 × 10^−6^/°C) was used to measure the internal pile deformations. Figure 2 shows the strain gauge attached with polyester resin to one side of the steel reinforcing bar, polished prior to installation. Traction tests were performed in the laboratory to calibrate the measured displacement on the reinforced steel bars.

These sensors were installed in two adjacent piles (piles 11 and 12) to measure longitudinal strains. Both piles were identical and in the center zone of the excavation, which was approximately 90 m long, where these piles were located. In pile 11, strain gauges were located every 2 m along the pile length, and every 4 m for the case of pile 12. Figure 1b shows a schematic cross-section with the location of strain gauges for pile 12.

The relation between length and electrical resistance in the strain gauge is given by

(1) $$GF=\frac{\Delta R\cdot L}{R\cdot \Delta L},$$

where *GF*: Gain factor; *R*: Original electrical resistance of the strain gauge (120 Ω); *L*: Original strain gauge length. An acquisition system SCXI (from National Instruments) of 16 bits of resolution and maximum sampling rate of 200 ks/S was used to obtain the data.

### 2.3. Optical Fiber

**Figure 3:**
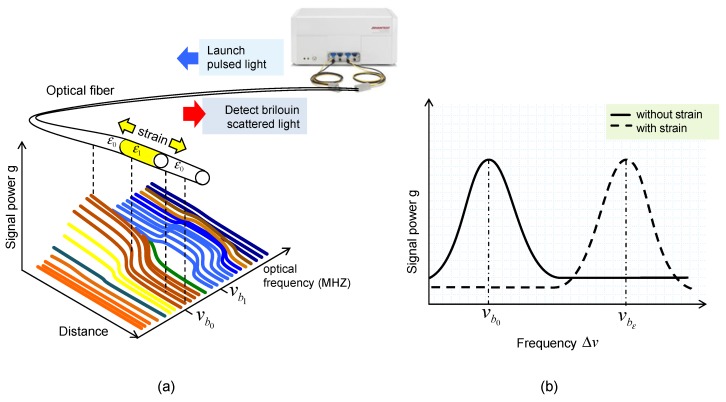
(**a**) Diagram of BOTDR equipment; (**b**) change in frequency with strain.

A Brillouin optical time-domain reflectometry (BOTDR) system from Advantest-NTT, as shown in Figure 3a, enables the longitudinal strain on optical fibers (software N8510) to be measured. Deformation in the longitudinal direction of the fiber generates a Brillouin frequency shift that is proportional to the fiber strain (see Figure 3b). The BOTDR equipment is able to measure the shift in Brillouin frequency and the time interval between launching pulse light and the received scattered light. Therefore, it can relate the deduced strain (every 5 cm along the fiber optic cable) to the longitudinal distance in the fiber where it was produced. Additional details of this technology can be found elsewhere in [14,15]. The measurement time lasts from 5 to 20 min; therefore, it does not allow dynamic data to be obtained. The same system allows measurement from meters to kilometers of distance. In the current setup, about 80 m of fibers was used and about 60 m was subjected to pile deformation. The sensing fiber was nylon-coated standard single-mode embossing optical fiber with a diameter of 0.9 mm, the instrument error was lower than 0.01% (derived from extension tests on the fiber and agrees with data from the manufacturer), and no compensation of temperature was applied during testing; however, the temperature was measured at different dates and depths, and it was found to be basically constant after the curing of concrete (difference below 3 m of depth lower than 1.5 °C). The maximum axial strain was recorded as always lower than the elastic strain limit of the fiber (1%). Additional details of the system and examples of measurements can be found in [16,17].

**Figure 4:**
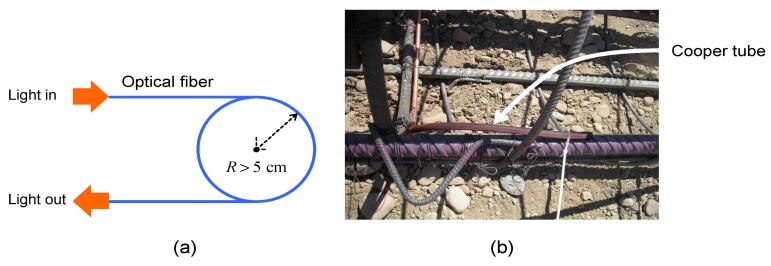
(**a**) Minimum curvature for the optical fiber scheme, (**b**) copper tube that gives the curvature, protects the fiber, and allows the fiber to start the loop to return to the surface.

The optical fiber was attached to the steel bar using plastic cable tie, and this was a fast and easy task on the field. The bond strength between the optical fiber and concrete developed as the concrete strength increased over time. The optical fiber was not pre-tensioned during installation; however, the curing of the concrete caused an initially low pre-tension. Fiber was installed continuously on a single pile cage that was placed carefully to avoid rotation during installation. As light traveled in the fiber interior, a minimum curvature radius was considered with the optical fiber, as shown at the bottom of the pile cage in Figure 4. This was done using a copper tube that gives the curvature, protects the fiber, and allows the fiber to start the loop to return to the surface (see Figure 4b).

The effects of soil excavation in pile strains were obtained by subtracting the base or initial measurement from the fibers to any measurement performed on other specific dates. This means that net strains are calculated by subtracting each recorded measurement of the strain values in the vertical or original configuration of the wall before excavation. This difference in the observed axial strain values for each stage of excavation is the key feature to back-analyze the measured strains and estimate the horizontal displacement of the wall.

### 2.4. Topography Survey

**Figure 5:**
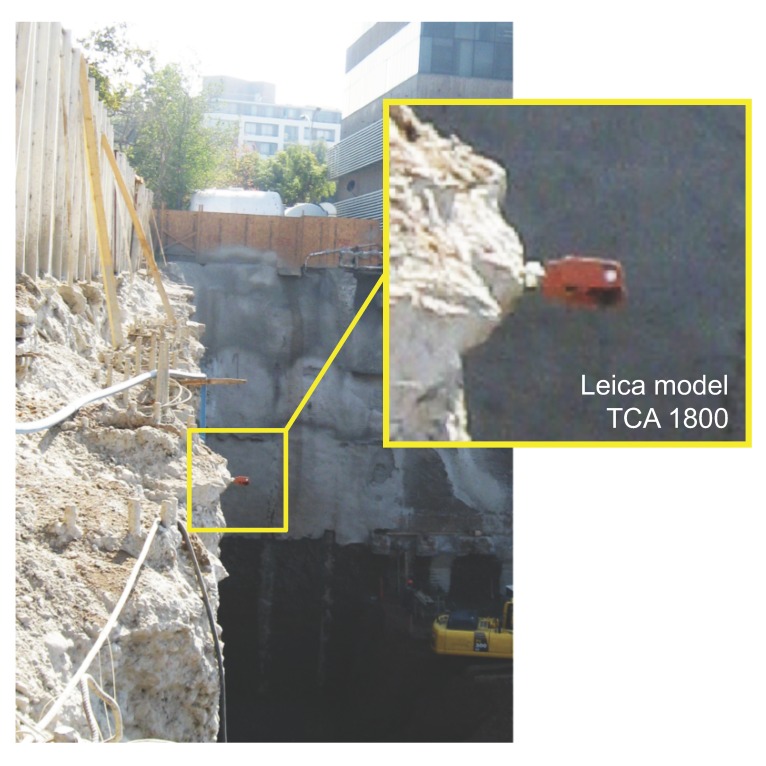
Prism used for geodesic measurement (Leica model TC1800).

The survey equipment used was a total station Leica model TC 1800 [18] with an angular precision of 2.54 cm. A prism was located in the top part of the pile, as shown in Figure 5. Horizontal distance measurements were obtained using 2 stationary reference points placed on fixed concrete survey markers located 20 m away from the excavation face. The distance from the total station to the prism located on the top of the pile, together with measurements of horizontal and vertical angles, allowed us to deduce horizontal displacements on the top of the pile.

## 3. Results

**Figure 6:**
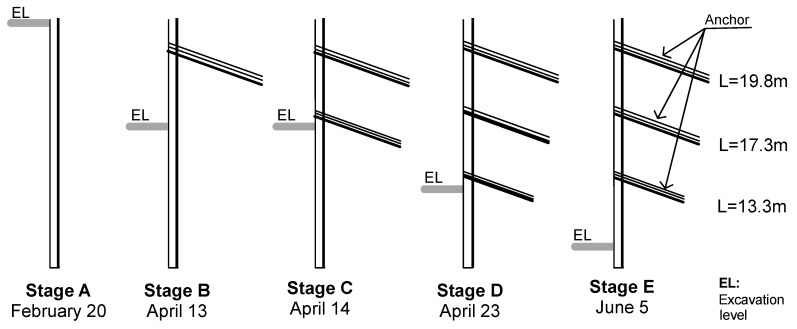
Excavation stages during monitoring using different sensors.

Measurements obtained from the strain gauges, optical fiber, inclinometer, and survey are presented in this section. Measurements were performed in different construction stages depending on availability due to safety concerns. Figure 6 presents a sketch of the different considered stages and shows the dates when these stages were reached.

### 3.1. Inclinometer Measurements

**Figure 7:**
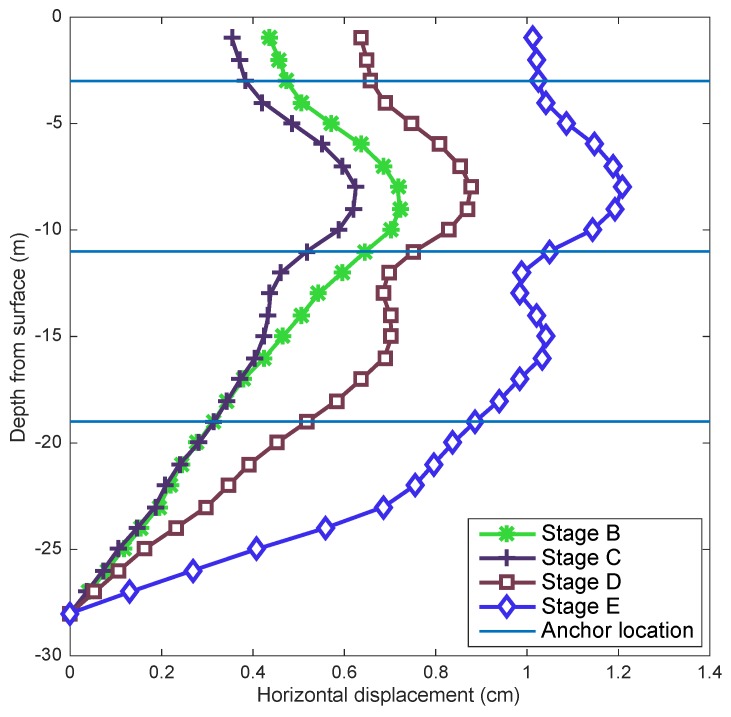
Pile horizontal displacements obtained from inclinometer measurements.

Inclinometer measurements were approximately performed every 1 m. From these data, it was possible to determine the pile horizontal displacement in the direction perpendicular and parallel to the wall. This was performed using the measured inclination angle and distance between measurements. The deduced displacement in the direction parallel to the wall was close to zero, and the same was observed with BOTDR measurements, as will be shown later. On the other hand, the horizontal displacements of the pile in the direction of the excavation are presented in Figure 7 for different stages. Measurements start from zero values at the bottom; however, this does not consider the possible movement of this point used as reference.

The maximum deduced horizontal displacement (with inclinometer) of the pile was 1.2 cm, as shown in Figure 7. However, these displacements correspond only to the flexure of the pile. If the pile also experiences rigid body movement, this displacement would not be detected by the inclinometer data as the inclinometer was completely embedded on the pile. In an extreme case, the pile could move horizontally a few centimeters (without flexure) without suffering any inclination along its length, and, therefore, the estimated displacements would be zero.

### 3.2. Optical Fiber Measurements

**Figure 8:**
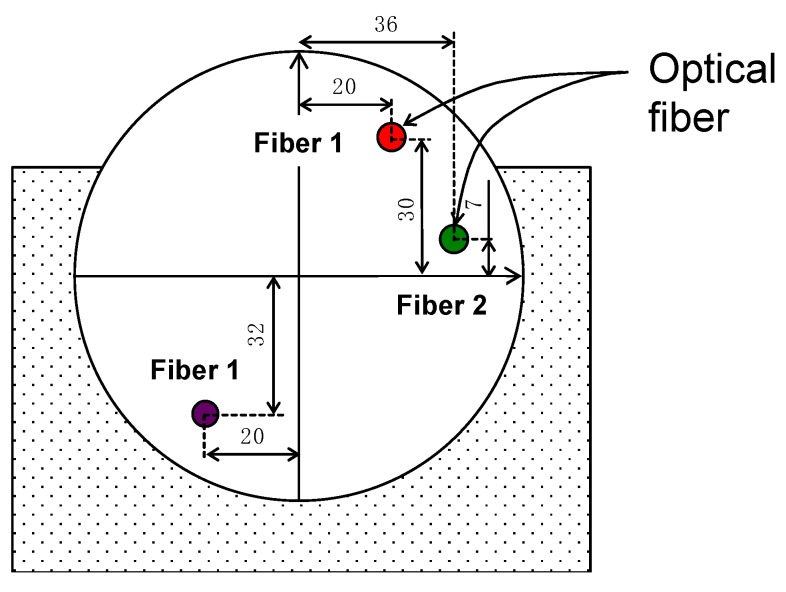
Pile horizontal displacements obtained from inclinometer measurements.

Figure 8 depicts the location of optical fibers in the pile cross-section. Three points are marked with the fiber location, fibers 1 and 2. Fiber 1 is marked twice as corresponding to the same fiber (going in downward and upward directions and using the copper tube described before). Fiber 2 is an independent fiber.

**Figure 9:**
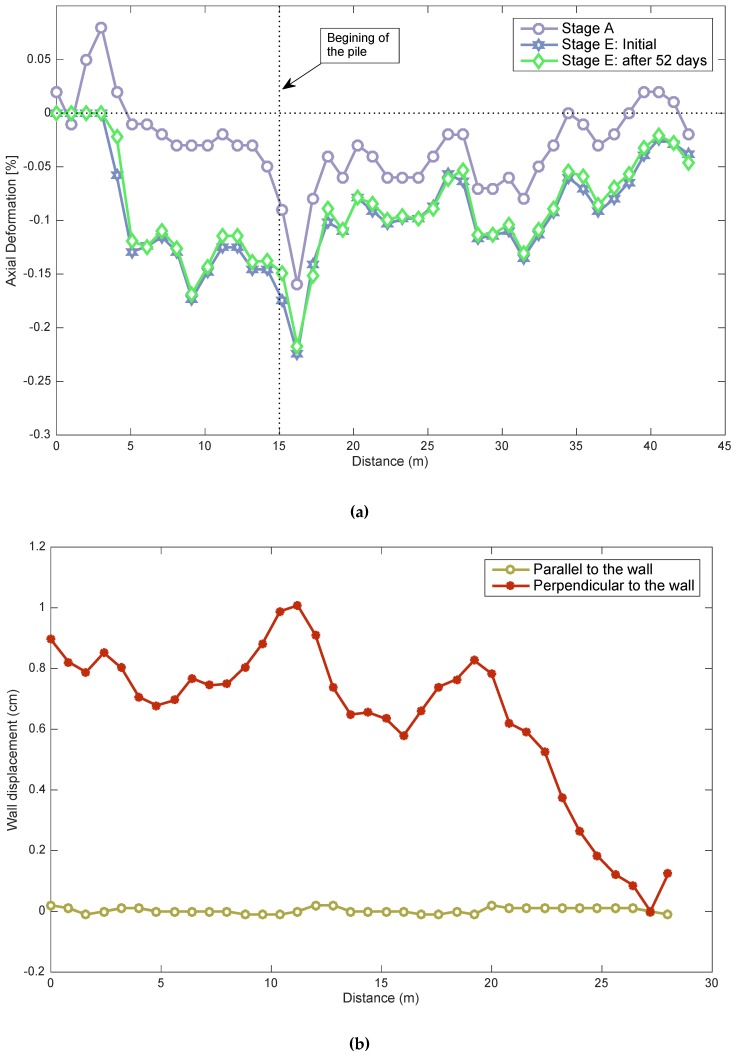
(**a**) Initial and stage E measurements of Fiber 1; (**b**) strain between initial and Stage E in perpendicular direction.

As indicated before, optical fibers were attached to steel bars in different locations (see Figure 4b). Figure 9a shows an example of measurements using the optical fiber (Fiber 2). The initial data (stage A) and the data for stage E along the pile where the optical fiber was attached are shown. The first 15 m of fiber corresponds to the fiber outside the pile wall and was, therefore, not considered in the analysis; the rest of the fiber corresponds to the fiber inside the pile. Similar measurements were obtained for fiber 1. The initial data show that the fiber was under compression, and this could occur due to concrete shrinkage during the setting/hardening process. To obtain the axial strain of the pile in each optical fiber, the initial measurements must be subtracted from the measurement of the stage analyzed. To deduce the horizontal displacement of the pile, strains deduced in each depth and its distance with respect to the center of the pile were considered and integrated along the optical fiber. In the final stage, two independent measurements are shown for two dates. It is possible to observe that the deformation on the pile-wall does not show changes after 52 days, as expected in gravelly soils, where consolidation is practically instantaneous. Moreover, there were no additional excavations or anchor tensioning during that period to cause a change in the pile-wall deformation. These two measurements also show good repetition of optical fiber data.

Figure 9b shows the deduced horizontal displacements perpendicular and parallel to the wall excavation face. The resulting displacements indicate that the movement parallel to the pile wall was negligible and that the one in the perpendicular direction was close to 1 cm.

**Figure 10:**
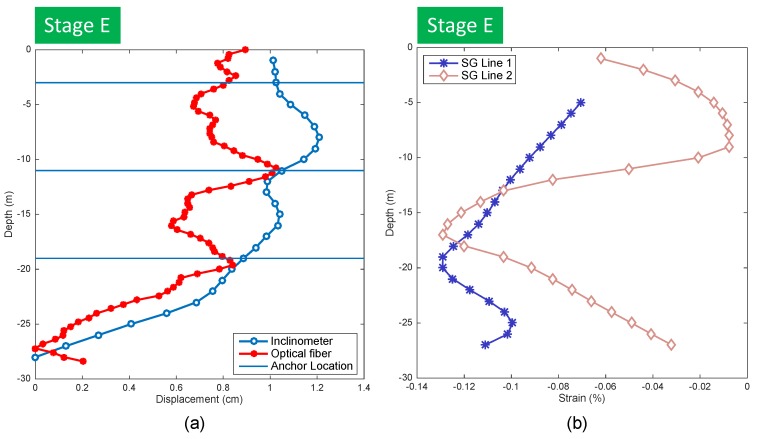
(**a**) Estimated displacements with optical fiber and inclinometers; (**b**) strain measured by optical fiber and strain gauges.

A comparison between estimated horizontal displacements from inclinometer measurements and optical fiber is shown in Figure 10a. It can be observed that they were similar in magnitude at some depths; however, the optical fiber sensor seemed to better capture the influence of the stressed anchor on pile deformation. As the length of the inclinometer probe did not allow measurements at distances less than 1 m, there was a notable difference close to the anchor position where drastic changes occurred due to tensioning caused by anchors.

### 3.3. Strain Gauge Measurements

About 50% of the strain gauges were not functioning properly mainly due to the damage they suffered during concrete casting. In this regard, the optical fiber and inclinometer showed a better behavior measuring without major inconveniences. Figure 10b shows two lines of strain gauge sensors and the variability observed on the obtained data.

In general, strain gauges showed compression, with values on the order of what would be expected. However, many (about 50%) of the strain gauges were malfunctioning; therefore, the data were disbelieved to be accurate and were not used to estimate the pile displacements. However, values measured from strain gauges, in general, allowed us to confirm that measurements obtained from the optical fiber and inclinometers were adequate, as they were of similar magnitude.

### 3.4. Topographic Survey Measurements

**Figure 11:**
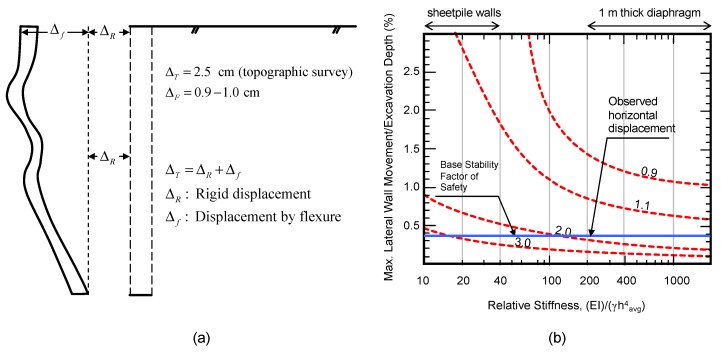
(**a**) Horizontal displacement measured in Stage E; (**b**) comparison between measured displacements and relationship adapted from Clough et al. [10].

A topographic survey performed between stages A and E gave a total horizontal displacement of 2.5 cm at the pile top. This value is about 1.5 to 1.6 cm larger than the values estimated from the inclinometer and optical fiber measurements (see Figure 10a). It seems like the difference was caused by a rigid body displacement of the pile wall that was not measured by the inclinometer and optical fiber, as these methods were not able to capture this type of displacement, because these were embedded completely inside the concrete pile (see Figure 11a).

The horizontal displacement measured during this monitoring program considers deformation that occurred during excavation after installation of the anchors. It is also deduced that there was an important component of deformation associated with the base of the excavation. Additionally, deformation can be considered instantaneous as no changes were observed at the same excavation stage after 1 or 2 months. The observed horizontal displacement (topographic survey) is plotted together with the relation proposed by Clough et al. [10], as shown in Figure 11b. The maximum horizontal displacement measured in this research is also included as a straight line at the bottom of this figure.

### 3.5. Numerical Modeling

Numerical simulations have been used in the past to reproduce the field-measured displacements [11,19], Bao et al. [20], and [7] evaluated soil-structure systems considering models that include nonlinear material and the geometry of both soils and structures. In this research, numerical studies were carried out using the software OpenSees [21] as an analysis platform. The wall pile model used nonlinear elements with fibers and force-based formulation [22,23]. The kinematic relation between strains and displacements was also nonlinear and approximated through a P-delta formulation. The model considered nonlinear uniaxial constitutive relations for concrete and reinforcing bar fibers. The Giuffré–Menegotto–Pinto [24,25] steel material object with isotropic strain hardening was used to model reinforcing bars.

This model is capable of representing the hysteretic behavior of steel reinforcement, exhibiting the Bauschinger effect together with isotropic strain hardening. Expected yielding stress (480 MPa) was used instead of the nominal value (420 MPa) for nonlinear analysis. A uniaxial model [26,27,28,29] was considered for the concrete with degraded linear unloading/reloading stiffness according to the work of Karsan and Jirsa [30] and no tensile strength. The concrete material was considered unconfined. The peak of the concrete constitutive relation was reduced to account for differences between the in-place concrete strength and standard cylinder compressive strength *f^’^_c_* (25 MPa for this case). For large-scale columns common in building construction, a factor C = 0.85 is recommended by Moehle [31]. This value, widely adopted in current building codes, was considered for this analysis. Truss elements with an initial stress material were used to model the post-tensioned steel cables. The initial tension force in the cables was 600 kN. The slip in the cables’ active zone was accounted through uniaxial elastic springs of stiffness 6 kN/mm (bottom and middle cables) and 1.5 kN/mm (top cable). Soil-pile interaction was modeled with gap elements (linear elastic behavior in compression and no tension strength). The soil ballast coefficient, used to calculate the gap stiffness in compression, ranged from 6.2 to 25 kg/cm^3^ close to the pile base. The relevance of the different factors that define the design of these pile walls in practice has also been evaluated.

**Figure 12:**
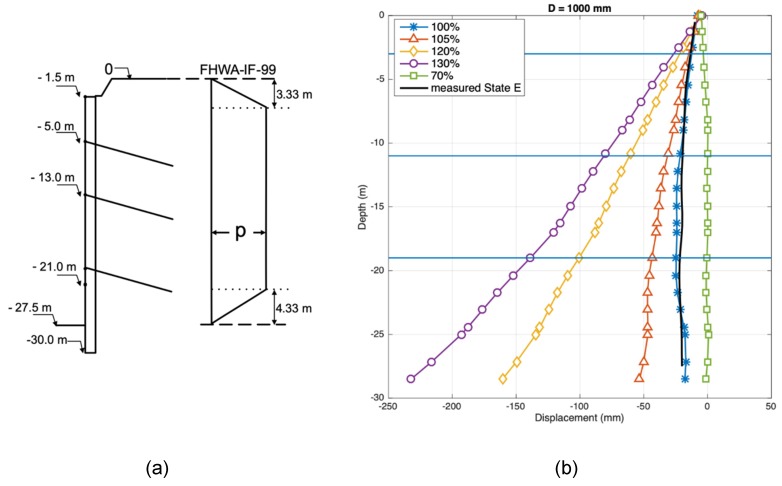
(**a**) Apparent earth pressure for granular material. (**b**) Horizontal displacement (dashed line: Measured on-site, continuous line: OpenSees model) over the height for different percentages of the specified value p in a 1000 mm diameter pile: 70%, 100%, 105%, 120%, 130%.

Figure 12a depicts the loads used to represent the earth pressure, obtained from [4]. The value *p* is obtained as

*p =* 0.65K_A_γh, (2)

where *K_A_* is the active earth pressure coefficient, γ is the soil unit weight, and *h* is the depth below the ground level. Considering typical measured parameters in Santiago gravel, *p* = 76.05 kN/m. A parametric study was performed to determine the influence of the *p* value on the expected displacements of the pile-wall. The pile-soil interaction is a very complex problem and the earth pressure distribution is, of course, a simplification to represent this behavior. Nevertheless, this approach is frequently used in the design of retaining walls and is shown to accurately represent the structural response measured on-site for this particular case.

Figure 12b shows the structural response of a 100 cm diameter pile for *p* = 76.05 kN/m. A sensitivity study to evaluate the dependence of the estimated displacements on the value of the parameter *p* was conducted. The range of considered values for *p* was 70%–130% of the specified value (76.05 kN/m). It is observed that the estimated displacements were very sensitive to variations in the specified earth pressure and, therefore, it is fundamental to have a good estimation of these pressures in practice.

Using a simple numerical model of the piles and the recommended p value (100%) gives a modeled response very similar to the actual observed value on the field using the optical fiber, inclinometer, and topographic survey.

## 4. Discussion

Different types of sensors have been used to measure the strains and displacement of the large pile-retaining wall on gravel. These sensors were installed during the construction of a real project keeping in mind the available time during the standard construction of this type of wall. Despite careful installation of all types of sensors (strain gauges, optical fiber, inclinometer pipes, and survey prism), strain gauges were shown to be the most difficult to successfully install with a relatively high number of sensors that do not deliver any data. The BOTDR optical fiber was shown to be very robust and the installation during construction was faster than the other methods. In addition, despite concrete being poured inside the piles, there was no major damage to the optical fibers, and the measurements responded successfully. The use of BOTDR optical fibers appears to be a good option to evaluate the performance of deep excavations. Careful interpretation of the data should consider that the movement of deep excavation has a rigid component that is not necessarily considered when measuring internal strains of piles/walls; an external measurement must be considered when analyzing the complete movement of these retaining systems.

It was shown in this project that BOTDR technology should be considered in monitoring structures involving soil and rock materials. It was revealed to be easy to install and have an adequate accuracy, even in actual construction conditions. It is advised to continue implementing this technology in geotechnical projects, always together with other types of instruments such as inclinometers.

Inclinometer measurements were also shown to be adequate, although, as the measurement depth interval was scarcer than the interval of measurements using optical fiber, the deduced horizontal displacement was less accurate and did not show the displacement due to the force applied to anchors.

Both inclinometer and optical fiber measurements, as they were done only inside the pile, did not show any rigid movement of the pile. A topographic survey was key in this case to find out that this portion of the total horizontal displacement was very important. This rigid body displacement is a function of the large strains needed by the soil at the bottom of the pile to reach its passive strength. In future projects, it is recommended to pass through the pile into the soil on its tip using either optical fiber or inclinometers. However, this task is not easy to implement during construction.

Numerical modeling using simplified design loads and guidelines agreed with the measured displacements for this project. Adequate measurements of the strains and displacement allow us to check how suitable current design methods are in civil and geotechnical engineering. Field data enable us to improve the design methods and are, therefore, necessary for monitoring actual projects and publishing these data.

## 5. Conclusions

This paper presents a monitoring program of lateral movement and internal deformation on a 28.5 m pile wall on gravelly soil. A companion numerical model was developed to study the effect of apparent applied pressure on a numerical model on the deduced horizontal displacement on the retaining wall. The main findings are as follows:

- Displacements of the pile wall were mainly observed in the direction of the excavation. The total horizontal displacement measured with a topographic survey at the pile top was 2.5 cm.
- Inclinometers and optical fiber proved to be adequate to measure the deformation of the pile wall, but not useful for measuring rigid body displacement of the pile as a whole. The installation of both systems was relatively simple, and measurements were very stable.
- Deformation in the gravelly soil was instantaneous, as expected. After one week, increments in measured displacements were almost negligible.
- Strain gauge installation was difficult and slow. Close to 50% of the strain gauges did not work properly after pile construction. Even though the rest of the strain gauges showed similar strains in comparison to the optical fiber, they did not provide reliable data, as other instruments used on site.
- Horizontal displacements estimated from numerical modeling are very sensitive to the magnitude of earth pressure. Therefore, a good estimation is necessary to evaluate wall displacements.
- Displacements of the pile-wall have a rigid displacement movement and a flexural displacement movement. It is necessary to capture the complete movement to measure with an external sensor as it was, in this case, using the topographic survey. BOTDR measurements were the most stable, and the results better represented the expected movement of the pile.

## Appendix A

**Table A1:** Main characteristics of inclinometer casing.

Type	QC Casing 70 mm (Slope indicator)
Internal diameter	70 mm
External diameter	59 mm
Thickness	11 mm
Maximum pressure	16.5 bar
Maximum load	635 kg
Temperature range	−29 to 88 °C

**Table A2:** Main characteristics of inclinometer.

Probe length	710 mm
Probe diameter	25.4 mm
Probe weight	1.4 kg
Memory	> 1,000,000 readings
Displacement error	2 mm per 25m
Temperature rating	−40 to 70 °C
Data Resolution	0.005 mm per 500 mm
